# A Standardized Prospective Memory Evaluation of the Effects of COVID-19 Confinement on Young Students

**DOI:** 10.3390/jcm10173919

**Published:** 2021-08-30

**Authors:** Francesca Pisano, Giulia Torromino, Daniela Brachi, Agnese Quadrini, Chiara Incoccia, Paola Marangolo

**Affiliations:** 1 Department of Humanities Studies, University Federico II, 80133 Naples, Italy; francescapisano00@virgilio.it (F.P.); torromino.giulia@gmail.com (G.T.); daniela.brachi96@gmail.com (D.B.); 2 IRCCS Santa Lucia Foundation, 00179 Rome, Italy; agnese.quadrini@gmail.com (A.Q.); c.incoccia@hsantalucia.it (C.I.)

**Keywords:** COVID-19 confinement, prospective memory, working memory, psychological wellbeing

## Abstract

The restriction imposed worldwide for limiting the spread of coronavirus disease 2019 (COVID-19) globally impacted our lives, decreasing people’s wellbeing, causing increased anxiety, depression, and stress and affecting cognitive functions, such as memory. Recent studies reported decreased working memory (WM) and prospective memory (PM), which are pivotal for the ability to plan and perform future activities. Although the number of studies documenting the COVID-19 effects has recently blossomed, most of them employed self-reported questionnaires as the assessment method. The main aim of our study was to use standardized tests to evaluate WM and PM in a population of young students. A sample of 150 female psychology students was recruited online for the administration of two self-reported questionnaires that investigated psychological wellbeing (DASS-21), prospective, and retrospective memory (PRMQ). Subjects were also administered two standardized tests for WM (PASAT) and PM (MIST). We found increased anxiety, depression, and stress and decreased PM as measured by self-reports. The perceived memory failures agreed with the results from the standardized tests, which demonstrated a decrease in both WM and PM. Thus, COVID-19 restriction has strongly impacted on students’ mental health and memory abilities, leaving an urgent need for psychological and cognitive recovery plans.

## 1. Introduction

The coronavirus disease 2019 (COVID-19), caused by the novel severe acute respiratory syndrome coronavirus 2 (SARS-CoV-2), has become a global epidemic, posing a serious threat to public health throughout the world [1,2]. To contain and mitigate the speed of viral transmission, governments of many countries implemented extraordinary measures, including social isolation, limitation of mobility, and suspension of commercial, educational, and social activities [3]. Since then, the entire population has been forced to stay at home for long periods of time. Many adults have experienced changes in their work, financial, and personal situations. Children and adolescents have completely restricted social contacts with their peers and significantly limited their physical activities [4], causing major changes in their daily routine.

The social containment measures for which no one was prepared had a strong impact on mental and physical well-being [5,6]. The negative impact on mental health has been documented by a growing number of studies showing higher levels of anxiety, depression, and stress during the pandemic compared to the pre-COVID-19 emergency [7,8,9]. Social isolation, uncertainty due to prolonged school/academic closure, and lifestyle changes have also negatively affected young people [10,11,12]. Indeed, several studies have reported increased rates of anxiety, stress, and sleep disturbances in the young population [13,14,15,16]. Ahmed and colleagues [11] investigated through an online survey the mental health status of 1074 Chinese people due to the outbreak of the COVID-19 and the mass isolation. The authors showed that young people aged between 21 and 40 years were more psychologically vulnerable, with higher levels of anxiety and depression and a lower mental wellbeing compared to the pre-COVID-19 pandemic. Similarly, another cross-sectional and nation-wide survey of college students in China confirmed these results, revealing the prevalence of acute stress, anxiety, and depressive symptoms during the COVID-19 emergency [15]. In line with this evidence, Wathelet and collaborators [16] demonstrated that university students seem to be particularly susceptible to mental health problems. Among risk factors, the authors have identified gender—with females being more affected—precariousness, social isolation, and low quality of social relations.

In addition to psychological variables, the distortion of temporal perception is another relevant aspect that emerged during the pandemic [17,18,19,20]. Recently, Ogden [17,18] reported a distortion in the perception of time in a large population of the UK. Indeed, 80% of people reported the feeling that time flew faster or slower during the lockdown with respect to pre-COVID-19 time. However, while during the first lockdown, a slowdown in the perception of time was associated with older age, increased stress, reduced workload, and lower levels of social interactions, during the second lockdown, age, gender, and workload did not influence the relative speed of time. A slower perception of time was associated with greater depression, shielding, and greater dissatisfaction with social interactions. Similarly, Cellini and colleagues [20] investigated the impact of the Italian restriction measures on young adults’ daily habits, including sleep quality, digital media use, and the subjective experience of time perception. The results indicated a lower sleep quality associated with a higher level of depression, anxiety, and stress and a slower perception of time during the lockdown compared to prior the lockdown.

It is well known that the perception of time is related to prospective memory (PM) [21,22], which is the ability to remember and therefore to perform planned activities at some point in the future [23,24]. Remembering to collect laundry or remembering to switch off the stove after cooking are examples of PM tasks and illustrate the importance of this type of memory in daily activities. PM consists of two components: event-based and time-based intentions. In event-based tasks, the participants are engaged in an ongoing task, such as searching for words in a crossword puzzle, reading words, answering questions, or making lexical decisions. When a cue related to the intention appears (e.g., a face, an object), participants must remember the intention and therefore make some explicit and recordable response [25,26,27]. Thus, in the event-based memory, the planned activity is elicited by external environmental stimuli. For example, meeting the boss may serve as a reminder to ask to leave work early. These cues often promote automatic processing that facilitates remembering [28,29]. In contrast, a time-based PM task requires the intention to remember to perform a planned activity at a particular time or after a specific period of time has elapsed [25]. For example, remembering to attend a doctor’s appointment at 13:00. As such, time-based tasks do not rely on external cues but require more conscious and intentional processing [25]. To date, many studies on age-related PM performance have been carried out in the laboratory, showing that young adults generally perform better than older adults in PM tasks (for a review, see [30]).

Different theoretical models of PM have been proposed so far, some of which identify an interdependence between PM and working memory processes (WM). WM can be defined as the mental workspace responsible for the maintenance and temporary manipulation of information crucial for complex cognitive tasks, such as learning, language comprehension, reasoning, and also for planning future activities [31,32,33,34]. Most PM theoretical models argue that remembering to execute an intention requires an interaction between attention and WM. However, theoretical perspectives differ in their emphasis on top-down versus bottom-up processes, among which attention and WM may be involved proactively or reactively [35]. The Preparatory Attention and Memory Processes (PAM) proposes that strategic monitoring, including rehearsing an intention and trial-by-trial checking for cues, is always required to detect the occurrence of the PM cue in the environment [36,37,38]. Another prominent PM model is the Multiprocess Theory [29]. According to this view, spontaneous retrieval is characterized as a bottom-up, cue-triggered process that is introspectively experienced as a memory popping into mind. The Multiprocess Framework suggests that an intention is spontaneously retrieved when the PM cue is salient or focal. Recently, the Multiprocess Theory has evolved into the Dynamic Multiprocess View (DMPV, see [35,39,40,41]), according to which strategic monitoring and spontaneous retrieval are not mutually exclusive, but they might interplay dynamically to mediate performance on PM tasks. For instance, in the example “Karen has to remember to buy a bottle of milk on her way home from work”, once Karen has formed this intention in the morning, she is unlikely to check the intention during her working day due to the lack of opportunity to buy milk. However, the event of getting into the car and starting the journey home could stimulate the recovery of the intention to stop at the supermarket and buy a bottle of milk. After recovering the intention in this context, Karen might start monitoring the supermarket on her way home. Thus, the DMPV model states that PM is accomplished via the flexible interplay of both top-down and bottom-up processes [35,39,40,41]. According to this dynamic view, WM capacity effectively engages monitoring at the appropriate moment and disengages it when the cues are unlikely to appear [35]. Indeed, WM processes are supposed to be necessary to support monitoring and to update the information relevant to the appropriate time point in which the intention has to be executed in PM tasks [30]. Accordingly, in a group of twenty-one young participants, Fronda et al. [42] found that failures in PM tasks were significantly associated with the highest load in WM tasks. Moreover, their ability to retrieve the information was less accurate in time-based than in event-based tasks. These findings are consistent with the assumptions that WM is more involved in self-controlled retrieval, which characterizes time-based PM tasks [42,43,44].

Given the importance of PM in everyday life [30], it is essential to understand the functioning of PM in acute stress situations. Several works have shown that high levels of stress lead to an uncontrolled production of cortisol, which affects cognitive functioning [45,46], also altering PM in different ways (for a review, see [47]). Some research revealed an enhancement of time-based PM performance in stressed participants compared to controls [48,49,50,51], while others revealed a negative effect of stress in event-based PM tasks [52].

Considering the COVID-19 pandemic as an undeniably stressful event, it is reasonable to think that the confinement required during the pandemic might have affected cognitive processes. Indeed, recently, in a group of 1215 participants, Fiorenzato and colleagues [53] found a deterioration of attention and executive abilities during the lockdown period, showing an improvement in PM and retrospective memory (RM) compared to the pre-COVID-19 period. Given that work stoppage is one factor of cognitive vulnerability, the authors hypothesized that the participants’ memory skills did not deteriorate because most participants continued to work remotely from home.

In addition to PM, WM was also affected by the restrictive measures of the pandemic. As is well known, several studies have already shown that higher levels of anxiety are associated with poor WM performance [54,55]. In line with this evidence, during the first period of lockdown, Fellman and collaborators [56] confirmed a negative correlation between anxiety levels and WM performance, and Santangelo et al. [57] uncovered marked difficulties in memory and attention in a large sample of adults mainly constituted by non-workers.

To our knowledge, to date, most studies have used self-report questionnaires to assess the effects of COVID-19 on both psychological variables and cognitive skills. In the present study, we aimed at studying the impact of COVID-19 on psychological wellbeing, WM, and PM in a sample of 150 students by using standardized cognitive tests to reduce the effect of social desirability bias.

## 2. Materials and Methods

### 2.1. Participants

A total of 170 female students aged between 18 and 23 years old (mean = 19.8 years, SD = 1.37) with an educational level of 13 years were recruited for this study. All subjects attended the first year for their bachelor’s degree in psychology. Inclusion criteria were: Italian language as mother tongue; right-handed [58]; and no history of chronic or acute neurologic, psychiatric, or medical disease. In terms of geographic distribution, all participants came from Campania, a southern Italian region. During the confinement, all participants lived in two-parent families. Among the study sample, nobody was diagnosed with COVID-19. Of the initial 170 participants, 20 students dropped out for personal reasons. The final sample was therefore made up of 150 students.

G*Power 3.1 [59] was used to calculate the sample size with α  =  0.05 and a power = 80%. The analysis indicated that a total sample size of N ≥ 34 was necessary to detect a significant effect in our study. Our final sample comprised 150 students, which is a size well beyond this threshold.

### 2.2. Ethics Statement

The data analyzed in the current study were collected in accordance with the Helsinki Declaration and the Institutional Review Board of the IRCCS Fondazione Santa Lucia, Rome, Italy. Prior to participation, all participants signed an online informed consent form.

### 2.3. Materials

#### 2.3.1. Self-Report Questionnaires

The materials included two psychometrically self-report questionnaires:

(1) Depression Anxiety Stress Scales (DASS-21), which consists of 21 questions with 7 items for each scale (e.g., for anxiety, “I feel I am close to panic”; for depression, “I cannot experience any positive feelings”; for stress, “I find hard to wind down”); all subscales are rated on a four-point Likert scale ranging from 0 (never) to 3 (almost always). Higher scores indicate more severe emotional distress (max–min total score: 21–0) [60];

(2) The Prospective and Retrospective Memory Questionnaire (PRMQ), which consists of 16 items on a five-point scale (e.g., for prospective memory, “Do you decide to do something in a few minutes and then forget to do it?”; for retrospective memory, “Do you forget something that someone told you a few minutes before?”). Higher total scores indicate more frequent self-reported memory difficulties (max–min total score: 80–16) [61].

#### 2.3.2. Cognitive Tests

The Paced Auditory Serial Addition Task (PASAT) [62] and the Memory for Intentions Screening Test (MIST) [63] were used.

The PASAT measures WM performance. The test consists of a list of 60 numbers between 1 and 9. Subjects are asked to add up each auditorily presented number in the list with the next one and to give the response within 1.8 milliseconds. For example, if the first two numbers presented are 5 and 6, the subject must answer 11 (6 + 5) [62].

The MIST assesses the ability to remember and perform planned activities at some point in the future. It consists of 8 everyday PM activities that involve the individual in an assigned task at a specific time (i.e., after 2 min or 15 min from the assignment) or when a specific cue is given (i.e., an event cue or a time cue). For instance, for event-based tasks: “When I show you a red pen, write your name in the chatroom”; for time-based tasks: “In 15 min, please ask me to take a break”. Within the time frame in which subjects are required to remember to perform the assigned tasks, they are involved in an ongoing task (i.e., word search puzzle) [63].

### 2.4. Procedure

Prior to performing the experiment, each participant received all the information on the research protocol via an online information sheet. Subjects who agreed to participate in the study signed and sent the online informed consent.

Each participant logged into Microsoft Teams platform, which allows to share the visual and auditory stimuli of the PASAT and MIST tests between the experimenter and the participant for a duration of one hour by using a personal access code. The examiner, after verifying the number code, shared the computer screen. For the PASAT test, subjects had to respond within 1.8 milliseconds, while for the MIST test, participants had to answer within 2 min for items 3, 5, 7, and 8 and within 15 min for items 1, 2, 4, and 6. The cognitive tests, PASAT and MIST, measured the participants’ cognitive performance one month after the COVID-19 confinement (T1).

The two psychometrically self-reported questionnaires investigated the psychological factors (anxiety, depression, and stress) and prospective and retrospective memory by asking participants to compare their responses at two time-points: one month before (T0) and one month after (T1) the COVID-19 confinement. They were administered through a specific online platform (Google Forms, Google Inc., Mountain View, CA, USA).

The order of administration of the self-report questionnaires (DASS-21, PRMQ) and of the cognitive tests (PASAT, MIST) was randomized among participants.

### 2.5. Data Analysis

Data were analyzed with IBM SPSS Statistics 22 software. To verify the applicability of the parametric analysis, a Shapiro–Wilk normality test was applied, which did not reveal a normal distribution of the data.

For the self-report questionnaires, statistical analyses were performed on the mean percentage of responses with two separated Wilcoxon tests for DASS-21 and PRMQ to evaluate differences between T0 and T1 in anxiety, depression, and stress and in prospective and retrospective memory, respectively.

For the MIST and PASAT tests, we ran two separated Mann–Whitney U tests, which compared the mean number of incorrect—for the PASAT test—or correct responses—for the MIST test—at T1 with the normative data (T0) for the two tests. For the MIST test, the Wilcoxon signed-rank test for paired samples was also conducted to directly compare the results obtained in the event-based and time-based subtests at T1.

Finally, Spearman’s correlations were calculated to examine possible relationships within the self-reported questionnaires (DASS-21 and PRMQ) and within the standardized tests (PASAT and MIST) at T1. Only significant correlations are reported in the Results section.

For all analyses, *p*-values < 0.05 were considered as statistically significant.

## 3. Results

### 3.1. Self-Report Questionnaires

**Figure 1:**
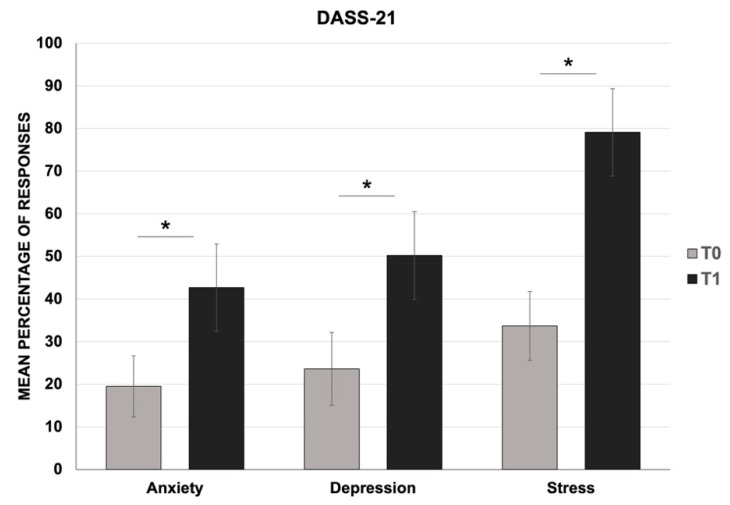
Mean percentage of responses for anxiety, depression, and stress scales (DASS-21) at two time-points: one month before (T0) and one month after the COVID-19 confinement (T1). * *p* < 0.001 T0 vs. T1.

For the DASS-21 test, the Wilcoxon signed-rank test revealed a significant increase in the mean percentage of anxiety (T0: mean = 19.5, SD = 7.2; T1: mean = 42.7, SD = 10.2; Z = −10.47; *p* < 0.001), depression (T0: mean = 23.6, SD = 8.5; T1: mean = 50.2, SD = 10.3; Z = −10.67; *p* < 0.001), and stress levels (T0: mean = 33.7, SD = 8.1; T1: mean = 79.1, SD = 10.2; Z = −10.65; *p* < 0.001) between T0 (pre-) and T1 (after confinement) (Figure 1). Moreover, the comparison of the mean score obtained at T1 for each domain of the DASS-21 to normative data confirmed an increased level of anxiety, depression, and stress, which reached the level of “severe” (Table 1).

**Figure 2:**
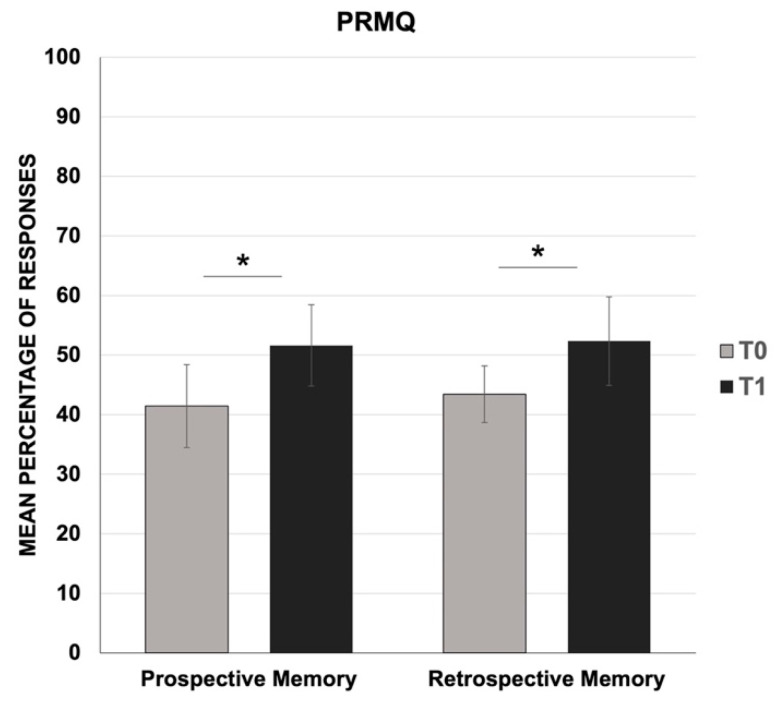
Mean percentage of responses for prospective and retrospective memory scales (PRMQ) at two time-points: one month before (T0) and one month after the COVID-19 confinement (T1). * *p* < 0.001 T0 vs. T1.

In parallel, the Wilcoxon signed-rank test revealed a significant increase in the mean percentage of responses for both the prospective (T0: mean = 41.4, SD = 6.9; T1: mean = 51.6, SD = 6.8; Z = −9.96; *p* < 0.001) and the retrospective memory (T0: mean = 43.5, SD = 4.8; T1: mean = 52.4, SD = 7.4; Z = −10.02; *p* < 0.001) (Figure 2), which suggests an overall increase in the participants’ self-reported failures in their memory abilities during the confinement. The comparison of the PM performance at T1 to normative data confirmed the above results (Table 2).

### 3.2. Cognitive Tests

**Figure 3:**
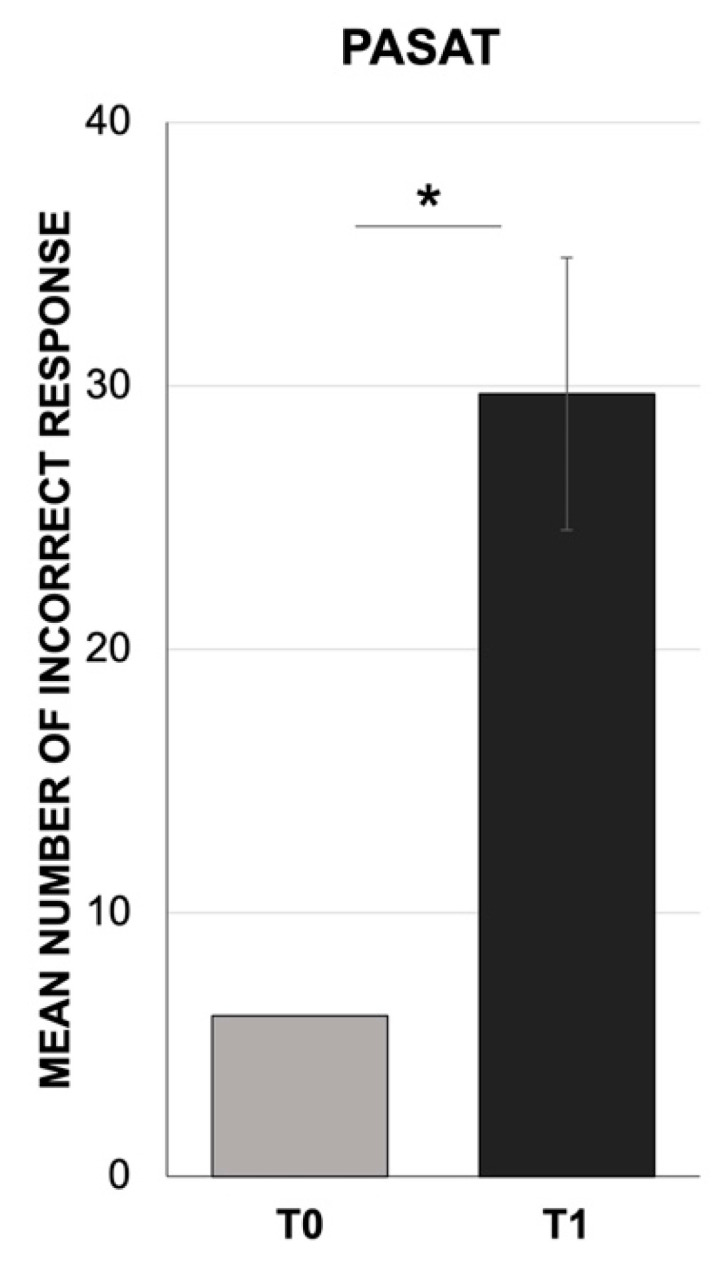
Mean number of incorrect responses for the working memory test (PASAT). The results collected at one month after the COVID-19 confinement (T1) compared to the normative data for the test (T0). * *p* < 0.001 T0 vs. T1.

**Figure 4:**
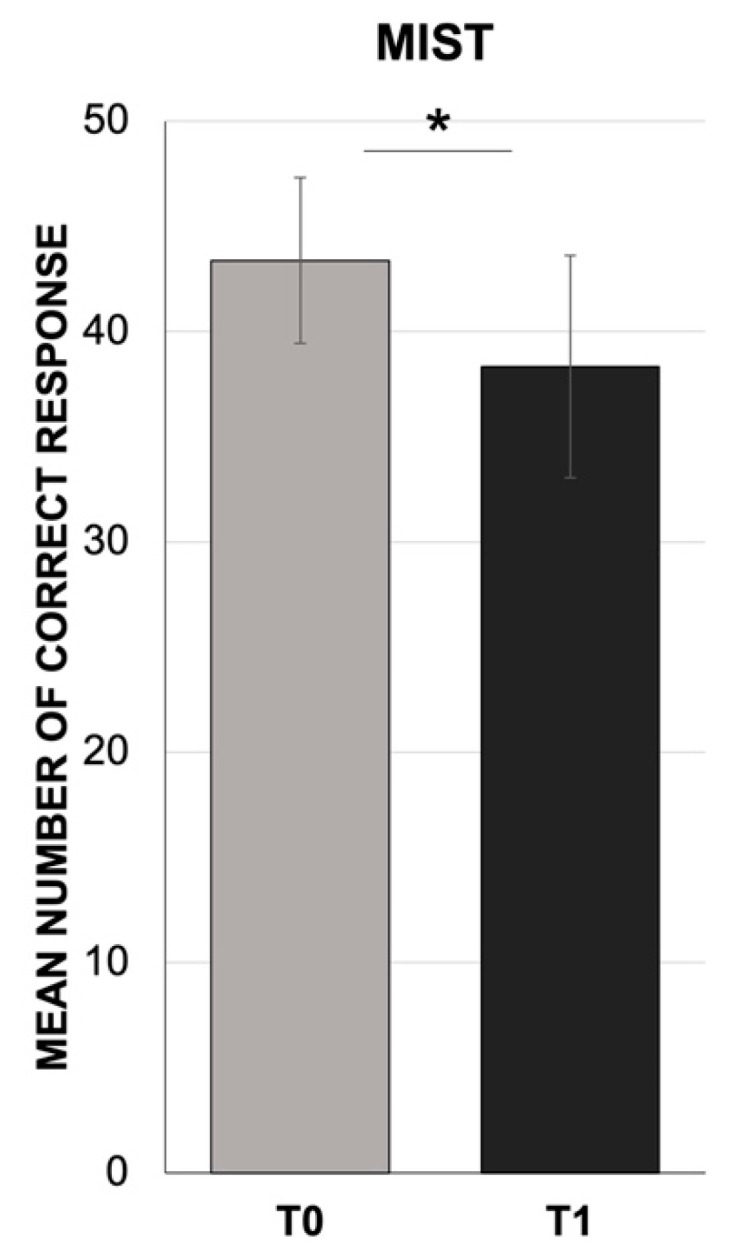
Mean number of correct responses for the prospective memory test (MIST). The results collected at one month after the COVID-19 confinement (T1) compared to the normative data for the test (T0). * *p* < 0.001 T0 vs. T1.

**Figure 5:**
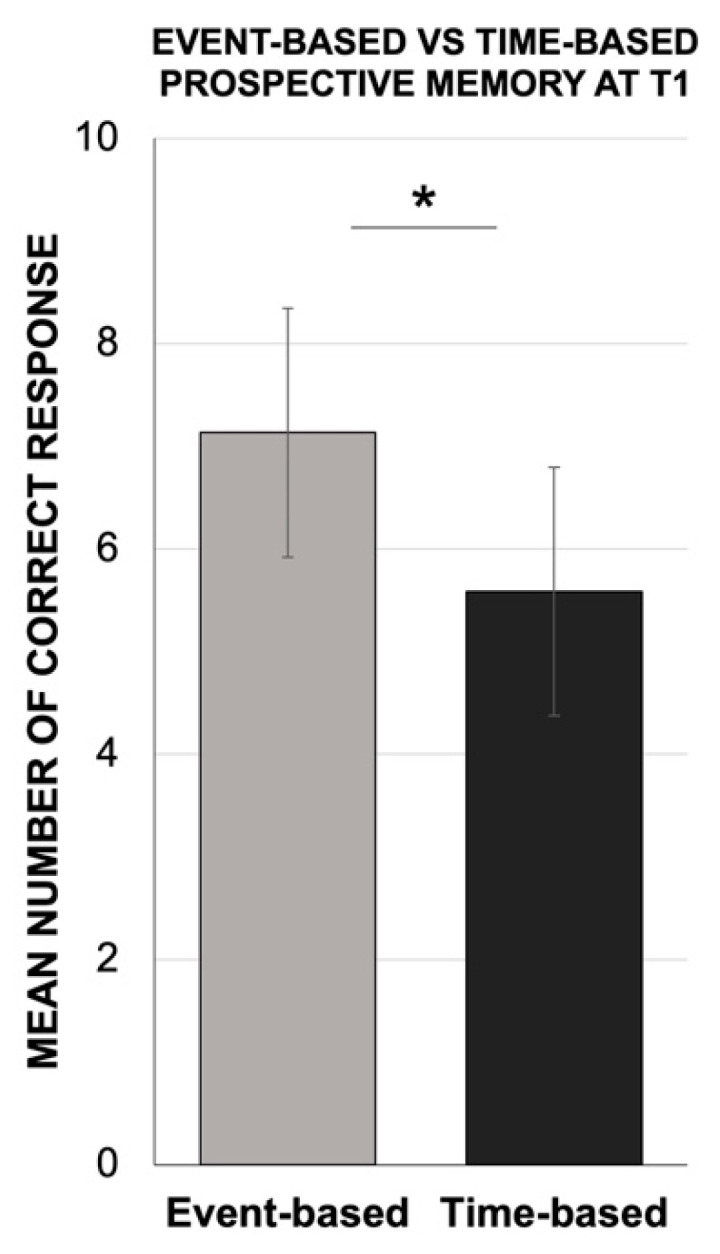
Mean number of correct responses for event-based and time-based tasks of the prospective memory test at T1. * *p* < 0.001 at T1.

In the PASAT test, the Mann–Whitney U test revealed a significant decrease in the participants’ performance (mean = 29.7, SD = 5.2; Z = −14.99; *p* < 0.001) at T1 compared to normative data (T0) [62] (Figure 3). Similarly, we found a significant decrease in PM (mean = 38.3, SD = 5.3; Z = −9.66; *p* < 0.001) by comparing the MIST results at T1 to its normative data (T0) [63] (Figure 4). In addition, the Wilcoxon signed-rank test showed a significant dissociation between event-based (mean = 7.1, SD = 1.2) and time-based (mean = 5.6, SD = 1.2) intentions in the MIST test, with lower scores in the time-based dimension with respect to the event-based tasks (Z = −8.12; *p* < 0.001) (Figure 5).

### 3.3. Correlation Analysis

A significant correlation between the anxiety levels, as measured by the DASS-21, and the participants’ performance in the PRMQ scale was found at T1 (Table 3). A significant correlation was also observed at T1 between the participants’ performance in the time-based tasks of the MIST and their WM performance measured through the PASAT (Table 4).

## 4. Discussion

The main aim of the present study was to investigate the impact of COVID-19 on psychological wellbeing, PM, and WM in a large sample of young students. For this purpose, unlike most of the previous studies, we used not only self-reported questionnaires but also standardized cognitive tests, which are less susceptible to social desirability and recall biases.

In line with several recently published studies [10,11,12,13,14,15,16], our results showed that our sample of students experienced increased levels of anxiety, depression, and stress at one month after the COVID-19 confinement compared to the pre-COVID-19 condition. These results were also confirmed when comparing our results to normative data, which, for all dimensions, revealed that our sample reached the level of “severe”. Thus, our data suggest that the pandemic has severely affected the mental health of our students, leading to social and emotional changes in their daily lives. Accordingly, to date, many studies have already pointed out that due to prolonged school/academic closure, lifestyle changes, and social distance, young people are more vulnerable to stress than older adults [65,66,67].

The findings from the self-reported PRMQ revealed that our sample perceived memory-domain failures during the confinement, both in the prospective and retrospective components, compared to the pre-lockdown period and to normative data. Thus, our findings are in line with previous suggestions, which proposed that lower rates of psychosocial well-being during the pandemic negatively affect the perception of time [17,18,19,20], a factor that is strongly related to PM processes [21,22]. In particular, some studies have identified stress as a detrimental factor for PM, suggesting that the psychological distress found in our students might have determined a distortion of time perception and, in turn, a PM disorder [52,68]. We also found a significant correlation at T1 between the score in the anxiety scale and the participant’s performance in the PMRQ questionnaire. Thus, not surprisingly, the perceived worsening in PM found in our sample was also influenced by its increased levels in anxiety.

Interestingly, the results in the MIST test confirmed a decrease in the participants’ PM performance compared to normative data, with a greater impact on time-based than on event-based tasks.

As reported in the introduction, PM involves two memory components: event-based and time-based intentions, which require different levels of cognitive demand and effort. Event-based tasks entail detecting cues or reminders in our environment related to previously established intentions (e.g., remembering to go to the supermarket for buying milk after work). These cues (e.g., a road sign referring to a supermarket) facilitate recall by promoting automatic processes [28,29]. Conversely, time-based intentions require the retrieval of previously formed plans (e.g., calling the doctor for a prescription) either at a specified time (e.g., at 6 p.m.) or after a certain time has elapsed (e.g., in 15 min). In this case, no external cues are provided that prompt the participant to initiate the performance. Thus, time-based intentions are cognitively more demanding than event-based intentions, as the former depend on implicit cues and require more self-initiation and monitoring [25,30]. Since confinement at home during the lockdown has significantly reduced the opportunity for students to take advantage of external cues to automatically recall future plans or intentions, a worsening in event-related memory was expected. However, we found that students had the worst performance in time-based tasks, which may be explained by the fact that these tasks require a greater cognitive load than event-based intentions, and they are known to depend upon WM engagement [42]. Indeed, together with a PM disorder, we found a decrease in WM performance, which was significantly correlated only with the time-based score of the MIST test. This latter evidence is in accordance with the most recent model of PM, the DMPV model, which posits that the ability to remember a planned intention depends upon the interplay between top-down and bottom-up processes, and it is influenced by WM capacity [35]. Accordingly, Fronda et al. [42] recently showed that time-based PM intentions are influenced by high cognitive load in WM tasks.

As far as we know, although previous studies have already shown a negative impact of the pandemic on different psychological and cognitive abilities [13,15,16,56,57], none of them measured the effects of the pandemic on PM tasks using standardized tests and their relationship with WM processes (but see [56] for WM). This constitutes the innovative and original aspect of our work with respect to most of the current studies conducted during the time of the pandemic, which included only subjective measurements. Indeed, in line with previous suggestions [69,70], we believe that the inclusion of standardized tests resulted advantageously since they are less influenced by response styles, social desirability, and self-report bias with respect to self-reported questionnaires. Accordingly, since the different tests rely on different ways to measure the subjects’ performance, as in the study by Arnold et al. [70], we did not find any significant correlations between the self-report scales and the standardized tests.

Thus, we believe that our results point to the urgency of using standardized tests to investigate the effects of the pandemic in different populations. These tests, although they were not administered before the pandemic, as no one could have predicted what happened, can still be considered as valid and reliable measures by comparing the results obtained during the pandemic to their normative data from large reference populations of different ages and educational levels. In fact, it is highly unlikely that the use of normative data in a large sample such as ours could have biased the results. We also believe that, in the near future, a possible way to overcome the difficulty in collecting data before the pandemic might be the development of longitudinal studies. Indeed, this approach might detect a stabilization of the observed changes in the cognitive and psychological domains between the different waves of the pandemic or instead an ability of the people to adapt themselves following the prolonged time of exposure to stress.

A possible caveat of our study is that the sample consisted of only young women from the Southern Italy. In fact, it was not possible to make a gender comparison between males and females. In the literature, several studies have shown that women are more vulnerable to anxiety, depression, and stress with respect to men [71,72,73]. For example, in a repeated cross-sectional study, in the early stages of the COVID-19 pandemic, Debowska and colleagues [71] showed a significant decrease in psychological well-being in female students from Poland compared to males. Moreover, the authors reported that young adult students (aged between 18–24 years old) had more symptoms of depression, anxiety, and suicidality than adult students (>25 years old). Similarly, in Essadek and Rabeyron’s work [72], female gender and younger age were identified as risk factors associated with mental distress during the COVID-19 pandemic. So far, no study has explored gender differences in PM tasks during the COVID-19 epidemic. To date, only the results of Fellman and colleagues [56] indicated that the impact of COVID-19 on WM cannot be explained by gender differences. It must also be considered that all of our students attended the first year for the bachelor’s degree in psychology; thus, our results cannot be generalized to all university students.

In conclusion, our data highlight the need for strategic plans to improve wellbeing, mental health, and cognitive performance in different populations and above all, in young people. Although in our study, we compared an actual population of students to normative data as a strategy to overcome the difficulty in measuring their performance before the beginning of the pandemic, we believe that the inclusion of standardized tests gives strength to our results, and it represents an element of novelty compared to previous studies on COVID-19 restrictions. As stated above and confirmed by previous studies [69,70], we believe that standardized tests are more sensitive to cognitive changes and less vulnerable to social desirability and recall bias compared to self-reported measures.

Before the pandemic, we were not prepared to prevent the emotional and cognitive effects we are still experiencing. Young people are more vulnerable to these effects. Indeed, the COVID-19 crisis has severely affected labor markets around the world, hurting young people more than other age groups. Globally, youth employment has fallen. Thus, the development of different recovery strategies and their implementation in schools and academics is an urgent need for our societies.

## Figures and Tables

**Table 1:** The mean scores of the anxiety, depression, and stress scale (DASS-21) at T1 compared to normative data [60].

Anxiety T1 Mean (SD)	Cut-Off
17.92 (4.3)	Normal 0–7
	Mild 8–9 Moderate 10–14 **Severe 15–19** Extremely severe 20+
**DEPRESSION** **T1** **Mean (SD)**	**Cut-Off**
21.08 (4.3)	Normal 0–9
	Mild 10–13 Moderate 14–20 **Severe 21–27** Extremely severe 28+
**STRESS** **T1** **Mean (SD)**	**Cut-Off**
33.21 (4.3)	Normal 0–14
	Mild 15–18 Moderate 19–25 **Severe 26–33** Extremely severe 34+

SD, standard deviation.

**Table 2:** The mean scores of the prospective memory scale (PRMQ) at T1 compared to normative data [64].

	Prospective Memory Mean (SD)	Retrospective Memory Mean (SD)
**T1**	20.7 (2.7) *	20.9 (3.0) *
**ND**	18.7 (5.5)	16.9 (5.0)

ND, normative data; SD, standard deviation. * *p* < 0.001.

**Table 3:** Spearman’s correlation coefficients (ρ) and their significance levels (*p*) are reported between the anxiety levels in the DASS-21 and the PRMQ-Prospective (PM) and Retrospective Memory (RM) subtest scores.

Self-Reported Questionnaire	Anxiety (T1)
**PRMQ-PM** **(T1)**	ρ = 0.1974 (***p* = 0.0154**)
**PRMQ-RM** **(T1)**	ρ = 0.2083 (***p* = 0.0105**)

**Table 4:** Spearman’s correlation coefficients (ρ) and their significance levels (*p*) are reported between the MIST total score, the event-based, and time-based tasks scores and the PASAT score.

Standardized Test	PASAT (T1)
**MIST (T1)**	ρ = −0.1139 (*p* = 0.1653)
**Event-based MIST (T1)**	ρ = 0.0112 (*p* = 0.8921)
**Time-based MIST (T1)**	ρ = −0.1970 (***p* = 0.0157**)

## Data Availability

The data presented in this study are available on request from the corresponding author. The data are not publicly available due to ethical and privacy restrictions.
